# Salicylic acid alleviates the effects of cadmium and drought stress by regulating water status, ions, and antioxidant defense in *Pterocarya fraxinifolia*

**DOI:** 10.3389/fpls.2023.1339201

**Published:** 2024-01-12

**Authors:** Hülya Torun, Bilal Cetin, Srdjan Stojnic, Peter Petrík

**Affiliations:** ^1^ Faculty of Agriculture, Düzce University, Düzce, Türkiye; ^2^ Faculty of Forestry, Düzce University, Düzce, Türkiye; ^3^ Institute of Lowland Forestry and Environment, University of Novi Sad, Novi Sad, Serbia; ^4^ Karlsruhe Institute of Technology (KIT), Institute of Meteorology and Climate Research-Atmospheric Environmental Research (IMK-IFU), Garmisch-Partenkirchen, Germany

**Keywords:** antioxidant enzymes, Caucasian wingnut, photosynthesis, reactive oxygen species, water scarcity

## Abstract

**Introduction:**

*Pterocarya fraxinifolia* (Poiret) Spach (Caucasian wingnut, Juglandaceae) is a relict tree species, and little is known about its tolerance to abiotic stress factors, including drought stress and heavy metal toxicity. In addition, salicylic acid (SA) has been shown to have a pivotal role in plant responses to biotic and abiotic stresses.

**Methods:**

The current study is focused on evaluating the impact of foliar application of SA in mediating Caucasian wingnut physiological and biochemical responses, including growth, relative water content (RWC), osmotic potential (Ψs), quantum yield (Fv/Fm), electrolyte leakage, lipid peroxidation, hydrogen peroxide, and antioxidant enzymes, to cadmium (Cd; 100 µM) and drought stress, as well as their interaction. Moreover, the antioxidant activity (e.g., ascorbate peroxidase, catalase, glutathione reductase, peroxidase, and superoxide dismutase activities) of the stressed trees was investigated. The study was conducted on 6-month-old seedlings under controlled environmental conditions in a greenhouse for 3 weeks.

**Results and discussion:**

Leaf length, RWC, Ψs, and Fv/Fm were decreased under all treatments, although the effect of drought stress was the most pronounced. An efficient antioxidant defense mechanism was detected in Caucasian wingnut. Moreover, SA-treated Caucasian wingnut plants had lower lipid peroxidation, as one of the indicators of oxidative stress, when compared to non-SA-treated groups, suggesting the tolerance of this plant to Cd stress, drought stress, and their combination. Cadmium and drought stress also changed the ion concentrations in Caucasian wingnut, causing excessive accumulation of Cd in leaves. These results highlight the beneficial function of SA in reducing the negative effects of Cd and drought stress on Caucasian wingnut plants.

## 1 Introduction

*Pterocarya fraxinifolia*, commonly known as the Caucasian wingnut, is a relict deciduous tree species that is a typical element of riparian and floodplain forests in lowlands of the Euxinian, Caucasian, and Irano-Turanian regions (Kutbay et al., 1999; Song et al., 2021). With its essential oil composition and high antimicrobial and antioxidant values, this plant is used as an antidiarrheal, poison, and dyeing agent by indigenous people (Akhbari et al., 2014; Tavakoli et al., 2016). Moreover, because of its high ornamental value, the genus *Pterocarya* has been utilized in urban landscape greening in recent years (Li et al., 2021). Though the Caucasian wingnut prefers moist soils, it is considered drought tolerant. However, there are a limited number of publications regarding this plant’s response to stress factors.

The detrimental impact of global warming and climate change on terrestrial ecosystems is manifested through the emergence of severe environmental conditions. Plants must contend with various stresses, and their survival is significantly compromised when exposed to combinations of multiple stress factors (Zandalinas et al., 2022). The frequency and intensity of abiotic stress combinations are predicted to increase (Zandalinas et al., 2022). Among these factors, soil-associated stressors such as water scarcity and heavy metals may be more detrimental in terms of plant morphological, physiological, and biochemical processes, which could lead to decrement in growth, biomass, and yield. Known as a highly toxic heavy metal, cadmium (Cd) is released into the environment through human activities such as mining, fertilizers, pesticides, and industrial processes (Tripti et al., 2023). Plants easily accumulate Cd from the soil, and excessive Cd uptake leads to metabolic defects and growth inhibition (Zhang et al., 2023a). Drought is another well-studied abiotic stress type that also causes serious effects on plant growth and survival (Ilyas et al., 2021). Given the coexistence of multiple stressors in the natural environment, simultaneous exposure to both drought and Cd stresses results in a decline in plant growth, development, and reproductive capabilities (Zandalinas and Mittler, 2022; Zandalinas et al., 2022). Although the physiological and biochemical responses of plants to drought and Cd stresses are well documented, both as single and combined applications (Bauddh and Singh, 2012; Xia et al., 2015; Adrees et al., 2020; Zhang et al., 2023a), there is still a lack of knowledge about the effects of these stressors on riparian and floodplain vegetation.

Acclimation to multifactorial stress conditions in plants is unique when compared to their response to each of the stresses applied individually (Zandalinas et al., 2021). Moreover, when plants face simultaneous abiotic stress, they need to cope with the combined effects of multiple stressors, which can be more challenging and have synergistic or antagonistic interactions (Zandalinas and Mittler, 2022). Drought and cadmium stress can stimulate the overproduction of both oxygen radicals and their derivatives, the so-called reactive oxygen species (ROS), such as hydrogen peroxide, hydroxyl, and superoxide radicals (Hasanuzzaman et al., 2020). Even though ROS are generated in the apoplast, chloroplasts, mitochondria, peroxisomes, and plasma membranes as part of normal cellular metabolism (Singh et al., 2019), the over-accumulation of ROS causes oxidative cell injury and can lead to damage of primary metabolites. To mitigate the harmful effects of oxidative stress induced by different stress factors, including drought and Cd, plants keep them under control by enzymatic (ascorbate peroxidase, catalase, dehydroascorbate reductase, guaiacol peroxidase, glutathione reductase, monodehydroascorbate reductase, and superoxide dismutase) and non-enzymatic (*α*-tocopherol, alkaloids, ascorbic acid, carotenoids, flavonoids, glutathione, and phenolic acids) antioxidant defense systems that help neutralize ROS and maintain cellular redox balance (Kerchev and Van Breusegem, 2022). The equilibrium between the production of ROS and the activities of antioxidant systems is vital for plant survival and the plants’ ability to withstand stress. Due to the dual role of ROS, plants have complex responses to stress conditions. However, there is no evidence that excess cellular levels of ROS induce significant damage in *P. fraxinifolia* under drought stress or Cd toxicity.

Salicylic acid (SA), a plant phenolic synthesized by the isochorismate or phenylalanine pathway in plants, is an endogenous phytohormone (Shine et al., 2016; Lefevere et al., 2020) that acts as a key signaling molecule in plant defense responses against various abiotic and biotic stress factors (Santisree et al., 2020; Song et al., 2023). As a plant growth regulator, SA interacts with other signaling molecules and phytohormones to reduce oxidative damage as well as modulate stomatal behavior (Prodhan et al., 2018; Li et al., 2023), reduce metal uptake (Sharma et al., 2020), scavenge ROS (Saleem et al., 2021), regulate nitrogen metabolism (Iqbal et al., 2022), and regulate various physiological and biochemical processes (Guo et al., 2019; González-Villagra et al., 2022; Kaya et al., 2023). However, the interaction between SA and abiotic stress in plants is a complex phenomenon and tends to vary depending on plant species, stress types, and environmental conditions (Liu et al., 2022). Therefore, understanding the role of SA in abiotic stress responses in woody plants is critical for developing strategies to enhance their resilience and productivity in the face of environmental challenges. Although several studies with SA-applied plant species have been reported, there are a limited number of studies regarding its application in simultaneously applied drought and Cd stresses. While previous studies have stated the alleviating impacts of SA on drought stress and Cd toxicity, the present study provides new insights into the mechanistic basis of this mitigation in woody plants. Furthermore, there is no conclusive evidence for the radical scavenging effect and antioxidant capacity of SA in Caucasian wingnut under drought or Cd stress.

To the best of our knowledge, there is no study investigating the effects of SA treatment on physiological and biochemical processes in *P. fraxinifolia* under drought and Cd stresses. Although agricultural plants are widely studied, studies on forest species are more limited. In addition, there are no publications on the drought and Cd stress tolerance of this species, either as a riparian species or in urban planting for its aesthetic value. Therefore, the purpose of this study was to assess the detrimental effect of single and combined treatments of drought and Cd stresses on Caucasian wingnut, as well as to investigate the potential contribution of SA to the reduction of oxidative stress. In this respect, leaf length, water content, osmotic potential, chlorophyll fluorescence, H_2_O_2_ content, lipid peroxidation, antioxidant enzymes, and ion concentrations such as Ca, Cu, Fe, K, Mg, Mn, P, Zn, and Cd, were measured in Caucasian wingnut seedlings grown under singly or simultaneously applied drought and Cd stresses. We hypothesize that SA application will alleviate drought and Cd stresses *via* upregulation of the antioxidant defense system (superoxide dismutase and glutathione reductase), compared to stressed non-SA-treated groups. Moreover, it is predicted that SA-treated groups will be able to maintain higher photosynthetic capacity (quantum yield) under stress treatments, compared to non-SA-treated groups.

## 2 Materials and methods

### 2.1 Plant cultivation and treatments

Caucasian wingnut (*P. fraxinifolia*) seeds were collected from seaside areas of the village of Uğurlu located in Akçakoca, Düzce Province (41°01′27.55″E, 30°59′36.37″N, 25 m a.s.l.) in October 2019. First, to remove any dirt or debris, the seeds were cleaned by rinsing them with distilled water. After cleansing, stratification was performed on these seeds at 4°C for 30 days. Then, the seeds were planted in plastic pots (9 cm × 11 cm × 20 cm) filled with a mixture of peat and perlite (at 75:25). The seedlings were grown in a greenhouse under controlled conditions: 27°C/16 h/day and 22°C/8 h/night at a relative humidity of 70%. After the seedlings were cultured for 6 months, the treatment groups were created as control (irrigation water alone; C), cadmium (100 µM CdCl_2_; Cd), drought (D), cadmium + drought (Cd+D), salicylic acid pretreatment (SA), salicylic acid pretreatment + cadmium (SA+Cd), salicylic acid pretreatment + drought (SA+D), and salicylic acid pretreatment + drought + cadmium (SA+Cd+D). A foliar application of SA was conducted, and 0.5 mM SA mixed with 0.1% Tween 20 was sprayed onto Caucasian wingnut leaves for 72 h before the stress treatments. The control plants were sprayed with an equal volume of water that contained only Tween 20 solution. Drought conditions were induced by maintaining soil water content below 40% of the full field capacity, and the drought treatment was considered to be leaving the plants without water. The concentrations were chosen on the basis of preliminary experiments in order to induce physiological processes without killing plants. Prior to harvesting, the plants were photographed, and the length of the leaves was measured using a standard ruler. Leaf length was defined as the distance from the base of the petiole to the apex of the terminal leaflet of the pinnate compound leaves of Caucasian wingnut. Mature leaves of this plant were harvested after 3 weeks of stress treatment, immediately frozen at −196°C, and stored at −80°C for further analysis. All chemicals used in this study were analytical-grade chemicals obtained from Sigma-Aldrich (St. Louis, MO, USA).

### 2.2 Relative water content

After harvesting, the fully expanded fourth leaves (n = 7) from each treatment were immediately weighed (FW). Then, the same leaves were floated in distilled water-filled tubes. Thereafter, they were reweighed to achieve the turgid weight (TW). Finally, the leaf samples were dried at 70°C until the dry weight (DW) was determined (Smart and Bingham, 1974). The relative water content (RWC) was calculated using the following formula:


RWC(%)=[(FW−DW)/(TW−DW)]×100=

### 2.3 Osmotic potential

After harvesting, the fully expanded fourth leaves (n = 7) from each treatment were utilized for the osmotic potential. First, a glass rod was used to crush the leaves of a Caucasian wingnut, and after centrifugation (5,000 *g* for 5 min), the supernatant was utilized for measurement using a vapor pressure osmometer (Wescor, Logan, UT, USA) (Santa-Cruz et al., 2002).

### 2.4 Chlorophyll fluorescence

Before harvesting, the fully expanded fourth leaves (n = 10) taken from each treatment group were used for the chlorophyll fluorescence measurements. First, the leaves were adapted to dark for 20 min with leaf clips, and then the measurements were performed using a chlorophyll fluorometer (Plant Efficiency Analyzer, Hansatech, Pentney, UK). The *Fv*/*Fm* ratio was measured for chlorophyll fluorescence to characterize the quantum efficiency of the photosystem II photochemistry.

### 2.5 Electrolyte leakage

Leaf disks excised using a cork borer from the fully expanded fourth leaves were utilized for the electrolyte leakage measurements (Lutts et al., 1996). Seven leaf disks from each treatment were washed with deionized water and then immersed in a test tube containing 10 mL of deionized water for 24 h at room temperature. After that, the initial electrical conductivity (EC1) of the samples was measured using a conductivity meter (Mettler-Toledo GmbH, Greifensee, Switzerland). The same test tubes were then autoclaved (120°C for 20 min) and cooled to room temperature. Then, a second electrical conductivity (EC2) measurement was taken. The EL was calculated using the following formula:


EL(%)=(EC1/EC2)×100

### 2.6 Lipid peroxidation

Lipid peroxidation levels of the Caucasian wingnut were determined by measuring thiobarbituric acid reactive substances (TBARSs). Leaf samples (0.5 g) were homogenized with 5 mL trichloroacetic acid (TCA; 0.1%) and centrifuged at 15,000 *g* for 10 min. After centrifugation, the supernatant (1 mL) was mixed with 4 mL of 20% TCA containing 0.5% thiobarbituric acid. The mixture solution was incubated at 95°C for 30 min. After the mixture had been cooled in ice, absorbance was recorded at 532 nm and 600 nm using a spectrophotometer (UV-VIS 1900i, Shimadzu Co., Kyoto, Japan), and the results were expressed as nmol/g FW (Heath and Packer, 1968).

### 2.7 Hydrogen peroxide

Hydrogen peroxide (H_2_O_2_) of Caucasian wingnut leaves was determined according to the method described by Liu et al. (2000). Leaf samples (0.5 g) were homogenized using TCA and centrifuged at 15,000 *g* for 10 min. After centrifugation, the supernatant (500 µL) was mixed with 1.5 mL of the reaction mixture containing 0.1% TiCl_4_. The H_2_O_2_ content was determined using a standard curve prepared on a spectrophotometer (UV-VIS 1900i, Shimadzu Co., Kyoto, Japan) at 410 nm, and the results were defined as l µmol H_2_O_2_/g FW.

### 2.8 Ion content

Dried Caucasian wingnut leaf samples (0.1 g) were extracted in 40 mL of 4% HNO_3_ to determine Ca, Cu, Fe, K, Mg, Mn, P, Zn, and Cd contents (Wheal and Palmer, 2010). The ion concentrations were measured using inductively coupled plasma–optical emission spectrometry (Avio 200 ICP-OES, PerkinElmer, Waltham, MA, USA).

### 2.9 Antioxidant enzymes

Frozen fresh leaf tissue (0.5 g) was homogenized in ice-cold 10 mL 50 mM KP buffer (pH 7.0) containing ethylenediaminetetraacetic acid (EDTA; 1 mM) and soluble polyvinylpyrrolidone (PVP; 1%) for antioxidant enzyme activity assays. Ascorbate (2 mM) was added to the same homogenization buffer for the ascorbate peroxidase (APX) activity assay. After centrifugation at 20,000 *g* for 15 min, the supernatants were obtained and further used for the enzyme activities and protein content assays. The protein content was assayed with bovine serum albumin (Bradford, 1976). Superoxide dismutase (SOD; EC.1.15.1.1) activity was measured at 560 nm according to the protocol described by Beauchamp and Fridovich (1971). The reaction solution contained 3 mL of 50 mM KP buffer (pH 7.0), 0.1 mM EDTA, 13 mM methionine, 0.075 mM nitroblue tetrazolium (NBT), and 0.002 mM riboflavin with the 50 µL enzyme extract. Then, the reaction mixture was kept under fluorescent light for 10 min. One unit of SOD was defined as the amount of enzyme that inhibited 50% of color reaction. Peroxidase (POX; EC.1.11.1.7) activity was measured at 470 nm for the activity increase with the oxidation of guaiacol (Mika and Luthje, 2003). The reaction mixture contained 3 mL of 25 mM sodium acetate buffer (pH 5.0), 10 mM H_2_O_2_, 10 mM guaiacol, and 50 µL enzyme extract. One unit of POX activity was defined as the amount that decomposes 1 μmol of H_2_O_2_ in 1 min. Catalase (CAT; EC 1.11.1.6) activity was determined at 240 nm for the absorbance activity reduction (Aebi, 1984). The reaction mixture contained 1 mL 50 mM KP buffer (pH 7.0), 10 mM H_2_O_2_, and 50 µL enzyme extract. One unit of CAT activity was defined as the amount that decomposes 1 μmol of H_2_O_2_ in 1 min. APX (EC 1.11.1.11) activity was measured at 290 nm for absorbance activity reduction (Nakano and Asada, 1981). The reaction mixture contained 1 mL 50 mM Na-P buffer (pH 7.0), 250 mM ascorbate, 5 mM H_2_O_2_, and 50 µL enzyme extract. One unit of APX was defined as the amount that oxidizes 1 μmol of ascorbate in 1 min. Glutathione reductase (GR; EC 1.6.4.2) activity was determined at 340 nm for the absorbance activity reduction (Foyer and Halliwell, 1976). The reaction mixture contained 1 mL of 50 mM Tris-HCl buffer (pH 7.6), 10 mM oxidized glutathione (GSSG), 5 mM NADPH, and 50 µL enzyme extract. One unit of GR was defined as the amount that reduces 1 μmol of GSSG in 1 min.

### 2.10 Statistical analysis

All treatments were performed with three independent biological replicates. The normal distribution of each trait was tested using the Shapiro–Wilk test, and the homogeneity of variances was analyzed by Bartlett’s test. In all figures, the error bars represent the standard errors of the means. The ANOVA and principal component analyses (PCAs) were conducted in the R statistical software v4.3.2 (R Core Team, 2022). A three-way ANOVA was used with drought, Cd, and SA as factors with fixed effects. PCA was conducted on scaled and centered data using the factoextra library (Kassambara and Mundt, 2020) to explore the overall relationship between the traits and treatments.

## 3 Results

### 3.1 Leaf length

**Figure 1:**
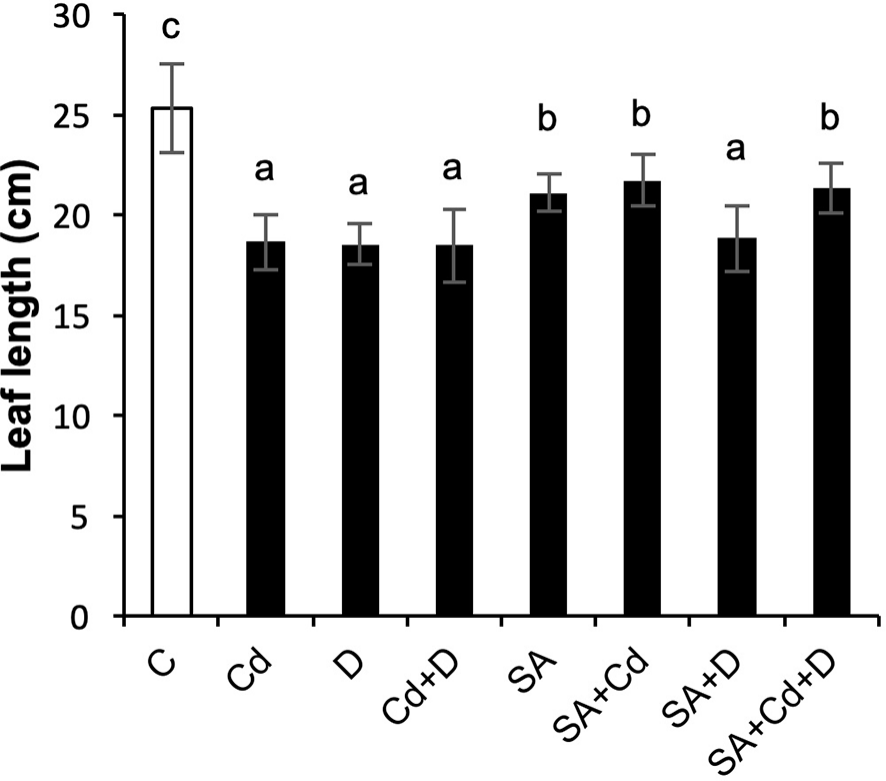
Effects of SA pretreatment on leaf length of *Pterocarya fraxinifolia* L. under drought, Cd, and the combination of these stress factors. Treatments: C, control; Cd, cadmium; D, drought; Cd+D, cadmium + drought; SA, salicylic acid pretreatment; SA+Cd, salicylic acid pretreatment + cadmium; SA+D, salicylic acid pretreatment + drought; SA+Cd+D, salicylic acid pretreatment + drought + cadmium. The bars (mean ± SD, n = 3) labeled with different letters are significantly different at *P* < 0.05 according to Duncan’s multiple range test.

**Figure 2:**
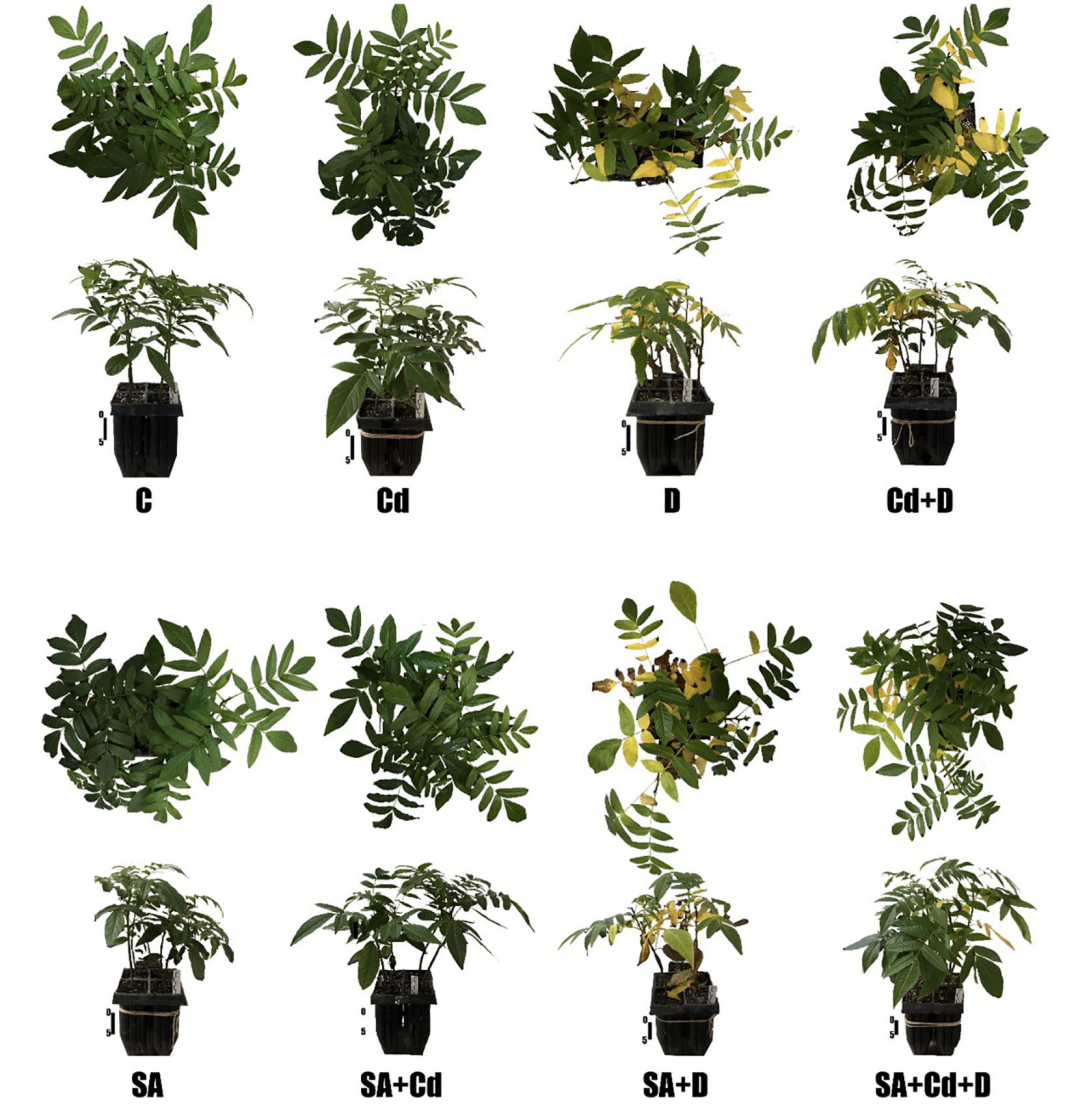
Effects of SA pretreatment on morphology of *Pterocarya fraxinifolia* L. under drought, Cd, and the combination of these stress factors. Treatments: C, control; Cd, cadmium; D, drought; Cd+D, cadmium + drought; SA, salicylic acid pretreatment; SA+Cd, salicylic acid pretreatment + cadmium; SA+D, salicylic acid pretreatment + drought; SA+Cd+D, salicylic acid pretreatment + drought + cadmium. Scale bar, 5 cm.

The growth of Caucasian wingnut exposed to drought or Cd stress as determined by measuring leaf length is shown in **Figure 1** and morphology in **Figure 2**. Drought and Cd stresses resulted in significant reductions in leaf length in this study. Plants subjected to drought stress, Cd stress, and combined application of these stress factors decreased the leaf length by 26.5%, 26.9%, and 26.9%, respectively. Interestingly, under normal conditions, SA caused a 16.6% reduction in leaf length. However, exogenously applied SA caused significant increases in plant leaf length by 16.7% and 15.1%, respectively, under Cd stress (SA+Cd) and drought and Cd stresses combined (SA+Cd+D) as compared to non-SA-treated plants.

### 3.2 Relative water content

**Figure 3:**
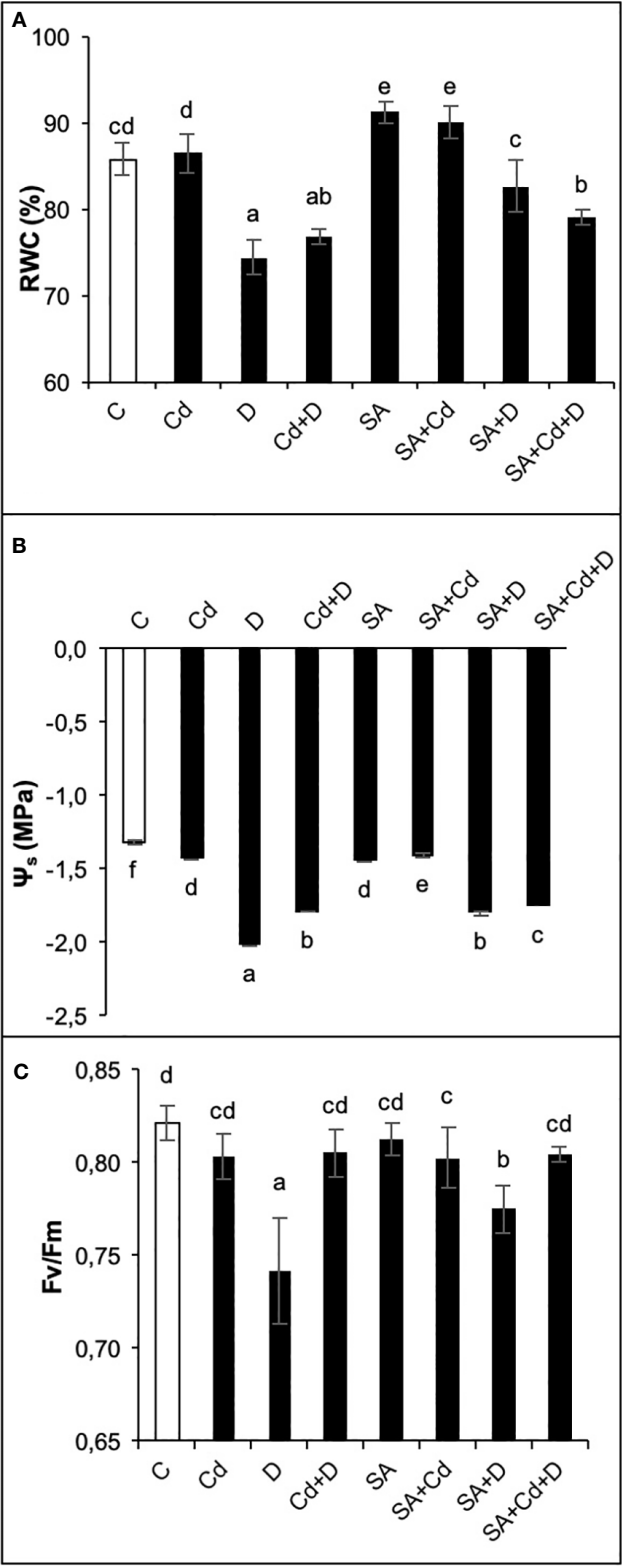
Effects of SA pretreatment on relative water content (RWC; **A**), osmotic potential (Ψ_s_; **B**), and chlorophyll fluorescence (Fv/Fm; **C**) of *Pterocarya fraxinifolia* L. under drought, Cd, and the combination of these stress factors. Treatments: C, control; Cd, cadmium; D, drought; Cd+D, cadmium + drought; SA, salicylic acid pretreatment; SA+Cd, salicylic acid pretreatment + cadmium; SA+D, salicylic acid pretreatment + drought; SA+Cd+D, salicylic acid pretreatment + drought + cadmium. The bars (mean ± SD, n = 3) labeled with different letters are significantly different at *P* < 0.05 according to Duncan’s multiple range test.

Drought stress alone and a combination of drought stress and Cd toxicity significantly (*p*< 0.05) reduced leaf RWC by 14% and 10.5%, respectively, compared to control plants (**Figure 3A**). Even though there was a reduction in RWC under stress conditions, it remained at 74% and 77% in D and Cd+D treatments, respectively. In contrast, no statistically significant effect of Cd stress was observed. Moreover, SA-treated control plants increased leaf RWC by 5.8% compared to non-treated control plants. In addition, RWC in SA-treated plants cultivated under drought and Cd stress treatments alone was respectively 4.7% and 12.2% higher than in non-treated plants subjected to the same stresses.

### 3.3 Osmotic potential

As expected, both single and combined applications of drought and Cd stresses significantly (*p*< 0.05) decreased leaf osmotic potential (Ψ_s_, **Figure 3B**). In comparison to the control group plants, the most pronounced reduction of leaf Ψ_s_ (35.5%) was in plants subjected to drought stress. Cd toxicity and Cd+D reduced leaf Ψ_s_ by 9.03% and 26.8%, respectively. Similar to leaf length, the application of SA led to a decrease in Ψ_s_ of 9.7% compared to the control treatment. In contrast, SA significantly enhanced leaf Ψ_s_ by 2.1%, 12.8%, and 1.7% in plants exposed to Cd, D, and Cd+D stress, respectively.

### 3.4 Chlorophyll fluorescence

Changes in photosynthetic efficiency (Fv/Fm) of the plants depending on the effects of Cd stress, drought stress, and their combination are shown in **Figure 3C**. No significant (*p*< 0.05) changes in Fv/Fm were detected by the applications of Cd and Cd+D stress, whereas the application of drought stress alone reduced Fv/Fm by 9.6%, as compared to non-stress-treated plants. Similar to this trend, plants grown under Cd and Cd+D treatments did not exhibit significant changes in Fv/Fm after exogenous application of SA. However, SA increased Fv/Fm by 4.4% under drought stress alone.

### 3.5 Electrolyte leakage

**Figure 4:**
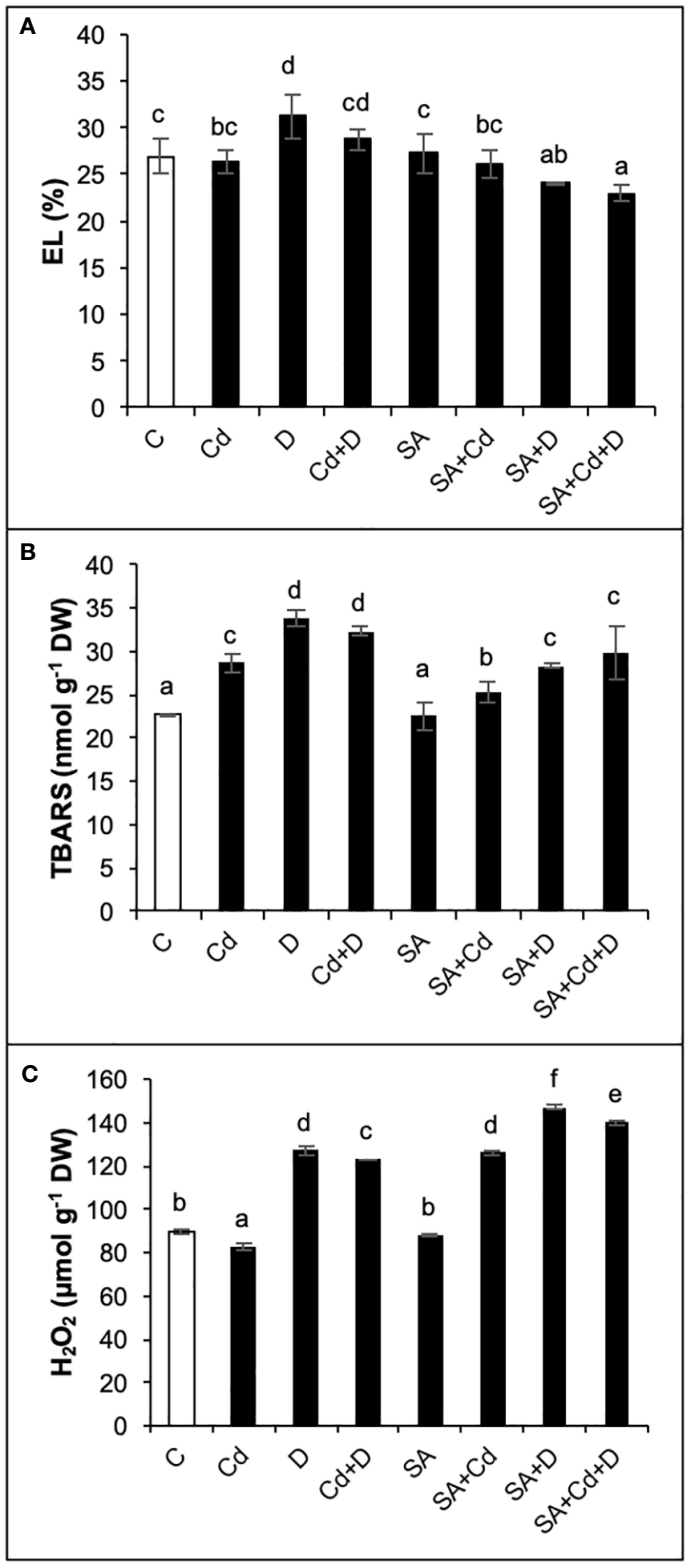
Effects of SA pretreatment on electrolyte leakage (EL; **A**), lipid peroxidation (TBARS; **B**), and hydrogen peroxide (H_2_O_2_; **C**) of *Pterocarya fraxinifolia* L. under drought, Cd, and the combination of these stress factors. Treatments: C, control; Cd, cadmium; D, drought; Cd+D, cadmium + drought; SA, salicylic acid pretreatment; SA+Cd, salicylic acid pretreatment + cadmium; SA+D, salicylic acid pretreatment + drought; SA+Cd+D, salicylic acid pretreatment + drought + cadmium. The bars (mean ± SD, n = 3) labeled with different letters are significantly different at *P* < 0.05 according to Duncan’s multiple range test.

As presented in **Figure 4A**, only drought alone significantly increased electrolyte leakage (by 15.6%) in comparison to controlled plants. In contrast, when comparing SA and non-SA treatments, the application of SA induced significant reductions in electrolyte leakage in D and Cd+D plants (22.8% and 20.5%, respectively).

### 3.6 Lipid peroxidation

Lipid peroxidation, which is evaluated from TBARS levels, increased by 26%, 48.9%, and 42.3% in Cd, D, and Cd+D treatments, respectively, compared to control plants (**Figure 4B**). The control plants did not exhibit any significant changes in TBARS content in response to SA pretreatment. Exogenous SA application displayed beneficial effects on lipid peroxidation and decreased TBARS levels by 11.9%, 16.3%, and 8% under Cd, D, and Cd+D treatments, respectively, as compared to non-stressed plants.

### 3.7 Hydrogen peroxide

As seen in **Figure 4C**, drought stress and the combination of drought and Cd stress treatment increased hydrogen peroxide (H_2_O_2_) levels by 41.5% and 37.2%, respectively, while a slight decrease in H_2_O_2_ (7.8%) was detected in Cd treatment compared to the control group of plants. There was no significant (*p*< 0.05) change in H_2_O_2_ after SA pretreatment. Contrary to TBARS, the exogenous application of SA increased H_2_O_2_ in all stress groups. Compared to non-SA-pretreated plants, the highest H_2_O_2_ accumulation (52.3%) was recorded in SA+Cd plants. Drought and combined stress also enhanced H_2_O_2_ levels by 15.9% and 13.9%, respectively, in comparison to the same group of plants subjected to SA.

### 3.8 Macro- and micronutrients

**Figure 5:**
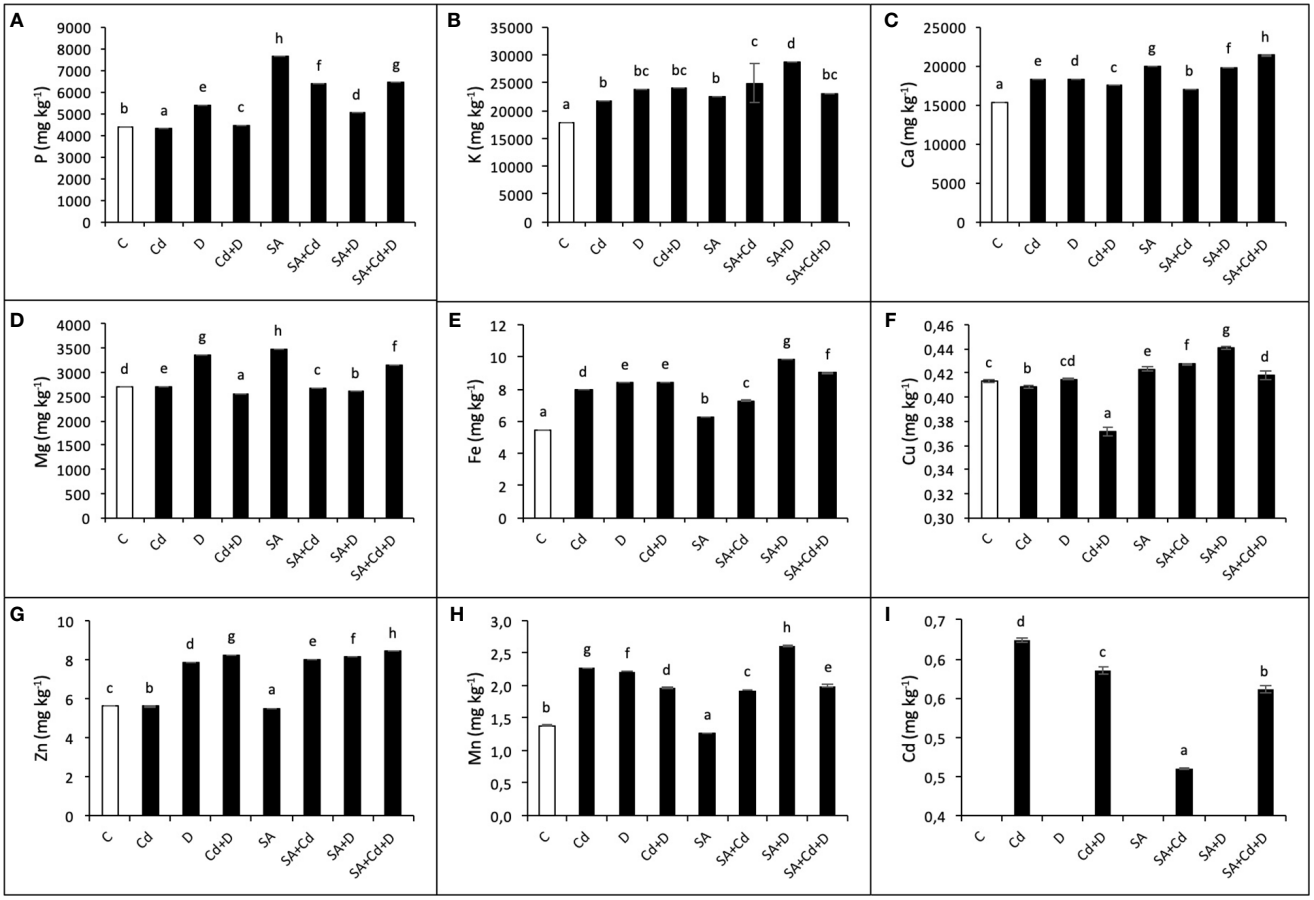
Effects of SA pretreatment on P **(A)**, K **(B)**, Ca **(C)**, Mg **(D)**, Fe **(E)**, Cu **(F)**, Zn **(G)**, Mn **(H)**, and Cd **(I)** of *Pterocarya fraxinifolia* L. under drought, Cd, and the combination of these stress factors. Treatments: C, control; Cd, cadmium; D, drought; Cd+D, cadmium + drought; SA, salicylic acid pretreatment; SA+Cd, salicylic acid pretreatment + cadmium; SA+D, salicylic acid pretreatment + drought; SA+Cd+D, salicylic acid pretreatment + drought + cadmium. The bars (mean ± SD, n = 3) labeled with different letters are significantly different at *P* < 0.05 according to Duncan’s multiple range test.

The effects of exogenous application of SA on macronutrient (P, K, Mg, and Ca) and micronutrient (Cu, Zn, Fe, and Mn) concentrations under stress conditions were investigated in the present study and are shown in **Figures 5A–H**. Drought stress, Cd stress, and their combination caused significant changes in the concentrations of macro- and micronutrients in Caucasian wingnut leaves. Under drought, among macronutrients, the highest accumulation was detected in K content (32.6%), and among micronutrients, in Mn content (60.9%), compared to control plants. Moreover, K, Ca, Fe, and Mn were enhanced in all stress treatments. P, Cu, and Zn displayed a slight decrease under Cd stress alone, while this reduction was recorded as 4.6% for Mg and 10.2% for Cu under Cd+D stress application when compared to non-stress-treated plants. Under non-stress conditions, exogenous SA pretreatment increased P (73.2%), K (25.4%), Ca (30.5%), Mg (29.2%), Fe (14.5%), and Cu (2.3%), while it reduced Zn (1.6%) and Mn (8%). In addition, compared to non-SA-treated plants, SA application under stress tended to increase nutrient levels, although not at a high rate. Under Cd toxicity, P, K, Cu, and Zn increased by 48%, 14.4%, 4.7%, and 43.5%, respectively, with exogenous application of SA. However, Ca, Mg, Fe, and Mn decreased by 7.3%, 1.1%, 7.6%, and 15.4%, respectively, under the same conditions. Only the P level decreased by 7% with SA pretreatment under drought (SA+D) as compared to non-SA-treated plants.

### 3.9 Cadmium content

Cadmium stress alone and Cd + drought stress significantly increased Cd levels (64.2% and 58.6%, respectively) compared to control plants (**Figure 5I**). In addition, there was no Cd accumulation in non-Cd-treated plants. In contrast, foliar SA application decreased Cd level by 26.1% and 4.2% in Cd and Cd+D treatments, respectively, when compared to non-SA-treated groups.

### 3.10 Antioxidant defense system

**Figure 6:**
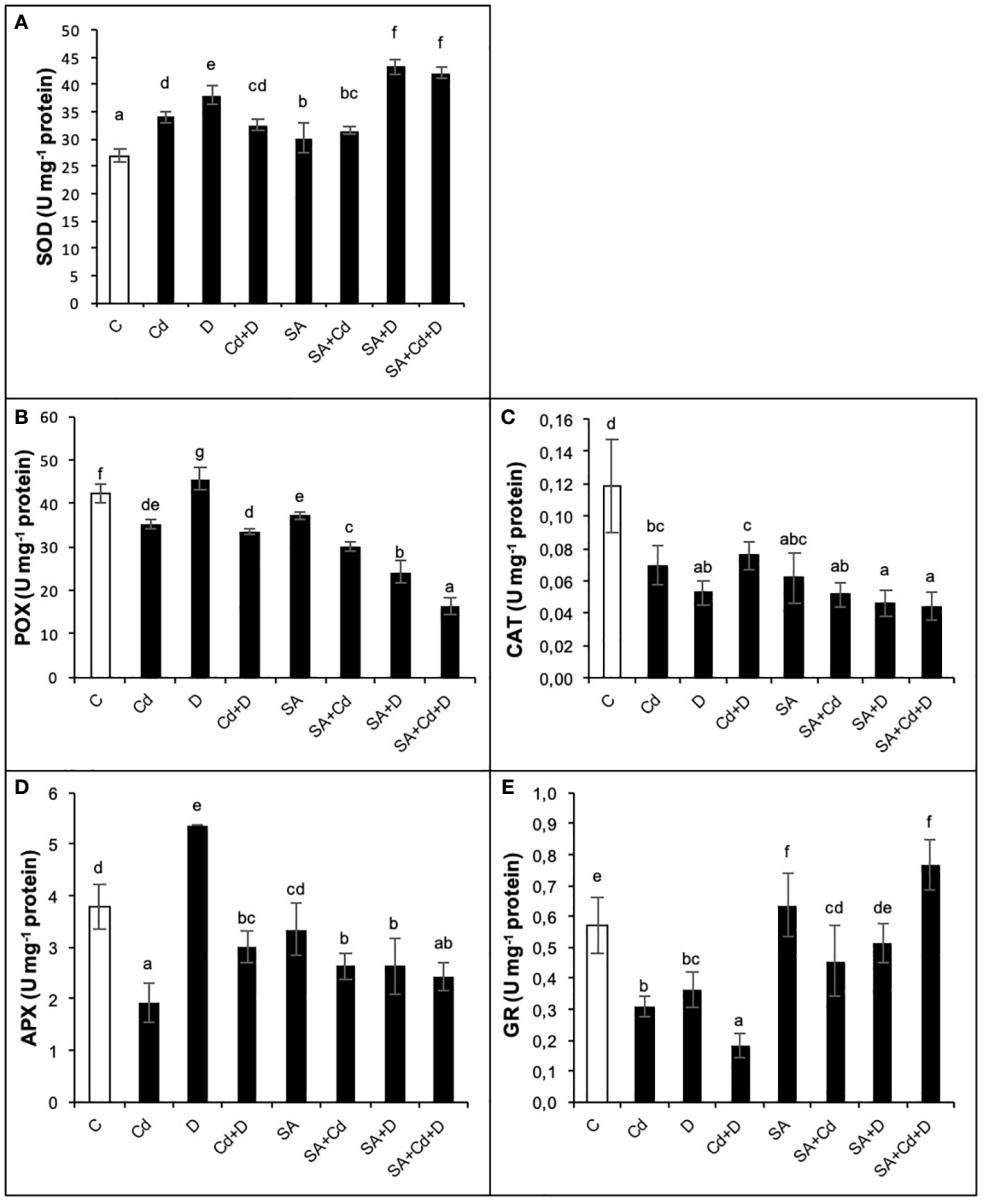
Effects of SA pretreatment on SOD **(A)**, POX **(B)**, CAT **(C)**, APX **(D)**, and GR **(E)** activities of *Pterocarya fraxinifolia* L. under drought, Cd, and the combination of these stress factors. Treatments: C, control; Cd, cadmium; D, drought; Cd+D, cadmium + drought; SA, salicylic acid pretreatment; SA+Cd, salicylic acid pretreatment + cadmium; SA+D, salicylic acid pretreatment + drought; SA+Cd+D, salicylic acid pretreatment + drought + cadmium. The bars (mean ± SD, n = 3) labeled with different letters are significantly different at *P* < 0.05 according to Duncan’s multiple range test.

As presented in **Figure 6**, Cd stress alone significantly decreased POX, CAT, APX, and GR activities by 16.7%, 41.7%, 50%, and 45.6%, respectively, while increasing SOD activity by 26.8%. Similarly, the combination of Cd and drought stress (Cd+D) lowered the same enzyme activities, whereas CAT activity rose by 21.2%. These reductions were detected as 19%, 33.3%, 21.2%, and 68.4%, respectively, under the combination of Cd and drought stress. Drought alone caused a significant (*p*< 0.05) increase in SOD (41.6%), POX (9.5%), and APX (42.1%) activities and decreased both CAT (58.3%) and GR (36.8%) activities as compared to non-stress-treated plants. Moreover, the activities of these enzymes, except CAT, were enhanced further after exogenous SA pretreatment in control groups. The CAT activity of Caucasian wingnut leaves was decreased by 50% with SA application as compared to control plants. Among the measured enzymes, a significant increase in GR activity was noted with the exogenous application of SA under all stress treatments. Cd stress, drought stress, and the stress combination enhanced GR activity by 48.4%, 44.4%, and 4.3 times, respectively, in plants pretreated with SA. In addition, drought and Cd alone did not cause a significant (*p*< 0.05) change in CAT activity with exogenous SA treatment, whereas a 50% reduction was recorded in SA+Cd+D plants. Furthermore, POX activity was reduced with Cd stress, drought stress, and their combination by 14.3%, 47.8%, and 52.9%, respectively, after SA pretreatment.

### 3.11 Principal component analysis

**Figure 7:**
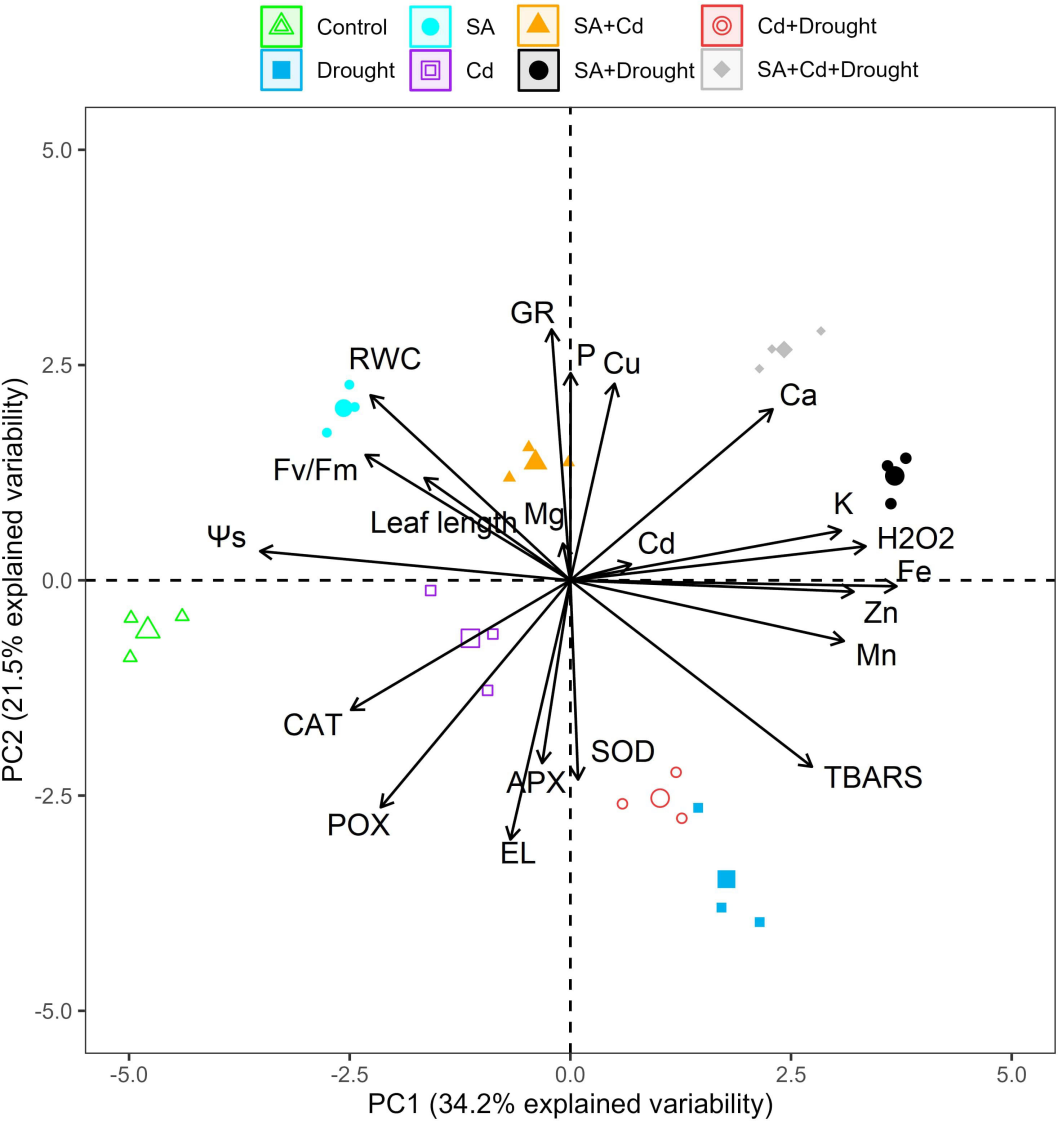
PCA bi-plots in the leaves of *Pterocarya fraxinifolia* L. under drought, Cd, and the combination of these stress factors. PCA, principal component analysis.

PCA was employed to study the variation in physiological and biochemical parameters obtained from the control seedlings and those treated with SA and Cd stress, drought stress, and their combination (**Figure 7**). The PCA extracted from the data measured in the current study (**Figures 1**, **3**–**6**) explained 55.7% of the total variance, where PC1 accounted for 34.2% of the variance and PC2 for only 21.5%. The analysis indicated that the macronutrients (K, Mg, and Ca) and micronutrients (Cu, Zn, Fe, and Mn), SOD activity, TBARS, H_2_O_2_ level, and Cd content of the Caucasian wingnut leaves were positively and significantly correlated (*p*< 0.01 or *p*< 0.05). SOD, H_2_O_2_, Cd, K, Ca, Cu, and Fe correlated strongly with the pretreatment of SA under drought stress alone (SA+D) and the combination of Cd and drought stress (SA+Cd+D) in the upper quadrant, but all other parameters (TBARS, Zn, and Mn) were grouped with the drought and Cd+D treatments in the right lower quadrant. Control, Cd alone, SA, and SA+Cd treatments and leaf length, RWC, osmotic potential, Fv/Fm, CAT, POX, APX, and EL were determined in the left upper and lower quadrants, with negative scores determined. These parameters were significantly (*p*< 0.01 or *p*< 0.05) negatively correlated.

## 4 Discussion

In the face of climate change, understanding how plants respond to more than one stress factor is critical, especially for woody plants, for developing strategies to maintain survival. Drought stress and Cd toxicity have detrimental effects on growth, membrane stability, and photosynthesis and cause osmotic and oxidative stresses (Hasanuzzaman et al., 2020; Iqbal et al., 2022; Zhang et al., 2023a). As a result of overproduction of ROS under these conditions, plants boost the antioxidant defense system with the activation of SOD, POX, CAT, APX, GR, ascorbic acid, glutathione, and secondary metabolites (Hasanuzzaman et al., 2020). Previous studies of the genus *Pterocarya* have investigated its growth performance, photosynthesis, physiological responses, and transcriptional changes in response to flooding and drought stress (Xu et al., 2015; Yang and Li, 2016; Li et al., 2021; Zhang et al., 2023b). However, to the best of our knowledge, no study to date has been conducted on the ROS regulation and antioxidant defense system of the *P. fraxinifolia* species under drought stress and Cd toxicity. Moreover, in the present study, we found that drought alone caused remarkably harmful effects on Caucasian wingnut plants compared to Cd treatment. This finding probably supports the finding of tolerance to the Cd presence of this woody species.

SA is a potential phytohormone that helps regulate plant growth and development by initiating various physiological and metabolic processes (Ding and Ding, 2020; Kaya et al., 2023). It additionally stimulates the overall antioxidant defense system at both enzymatic and non-enzymatic levels, which helps to remove excessive free radicals and alleviate the adverse impacts of abiotic stress factors (Liu et al., 2022; Kaya et al., 2023). Additionally, numerous studies have indicated that exogenous application of SA acts as a scavenger and enhances plant tolerance to drought stress and Cd toxicity by modulating the antioxidative enzymes (Guo et al., 2019; González-Villagra et al., 2022). In the present study, the function of exogenously applied SA in the regulation of growth, water content, chlorophyll fluorescence, mineral nutrients, lipid peroxidation, membrane integrity, and antioxidant enzymes under Cd stress, drought stress, and the combination of these stressors is elucidated.

In the current study, drought stress, Cd stress, and the combination of these stress factors caused a significant reduction of Caucasian wingnut leaf growth and water content. All stress treatments negatively affected leaf length, RWC, and Ψ_s_, with the most pronounced reduction observed in the drought treatment. This is in accordance with previous studies, which reported a significant decrease in plant growth and water content under drought stress, Cd stress, or their combination (Bauddh and Singh, 2012; Adrees et al., 2020; González-Villagra et al., 2022). Similar to this reaction, exogenous SA-treated Caucasian wingnut plants displayed better protection in growth and water content. Exogenous SA application had a beneficial effect by improving the leaf length, RWC, and Ψ_s_ in terms of the single and combined treatments of Cd and drought stress, which is in agreement with prior research conducted by Iqbal et al. (2022). When plants face drought or Cd stress, osmoregulation is achieved by the production of osmolytes such as amino acids, mainly proline, soluble sugars, and organic acids (Sharma et al., 2019). In addition, Zhang et al. (2023a) reported increased proline accumulation under the combination of drought and Cd stresses in subtropical coastal tree species. SA-mediated osmolyte synthesis may be the result of osmoprotection in which the Caucasian wingnut increases resistance under Cd stress, drought stress, and their combination.

Fv/Fm is one of the chlorophyll fluorescence parameters, representing the maximum quantum yield of PSII photochemistry. To understand the adaptation of plants to various environmental stressors, Fv/Fm can be used as an indicator of plant health (Force et al., 2003). Chlorophyll fluorescence content significantly decreased in Caucasian wingnut plants subjected to drought stress alone. Moreover, the Fv/Fm level remained above 0.803 and 0.805 in Cd- and Cd+D-treated plants, respectively, indicating that the PSII photosynthetic efficiency was well-protected under both stresses. Similar findings regarding drought-induced decreases in Fv/Fm have also been observed in *Acer* genotypes (Banks, 2018) and *Coffea arabica* (de Souza et al., 2020). The decrease in Fv/Fm under drought stress can be associated with damage to the thylakoid membranes and chlorophyll degradation. ROS accumulation and increments in SOD, POX, and APX activities recorded in the current study may be the result of decreased Fv/Fm when plants were exposed to drought stress. Similar to non-SA-treated drought-stressed plants, exogenous SA application altered Fv/Fm in our study. It has been observed that SA treatment in *Raphanus sativus* (Henschel et al., 2022) and *Zea mays* (Xin et al., 2023) can increase Fv/Fm under drought stress. Moreover, it has also been reported that exogenously applied SA also increases photosynthetic efficiency with a photoprotective effect (Moustakas et al., 2022).

A high accumulation of TBARS and H_2_O_2_ accompanied by an increase in EL under stress conditions triggers oxidative damage in cells due to their high capacity to move between biological membranes and thus disrupt the stability of the plasma membrane (Bienert et al., 2006). In the current study, Caucasian wingnut leaf H_2_O_2_ levels, peroxidation of membrane lipids, and electrolyte leakage increased under Cd stress, drought stress, and their combination (Abbas et al., 2018; Kabała et al., 2019; Adrees et al., 2020; Kaya and Shabala, 2024), indicating an increase in oxidative stress in drought- and Cd-treated plants. Interestingly, in this study, Cd toxicity alone did not significantly change the EL level, whereas TBARS content increased. In addition, H_2_O_2_ levels also decreased in Cd-treated Caucasian wingnut leaves. This result correlating with unchanged RWC and Fv/Fm may be associated with reduced ROS production under these conditions. This may have been a result of tolerance to Cd by this woody species. In addition, in this study, lipid peroxidation under Cd stress was not greater than that under drought stress alone or in combination. These results suggest that the detrimental effect of stress combination on membrane lipids was greater than that of Cd stress alone, but not that of drought alone. In our study, TBARS content and EL were alleviated by foliar application of SA at a level even less than the control level. Similar conclusions have been reported in research on *Solanum tuberosum* (Li et al., 2019), *Brassica juncea* (Iqbal et al., 2022), and *A. chilensis* (González-Villagra et al., 2022). The results displayed that TBARS and EL were suppressed by exogenous SA pretreatment under Cd and drought stresses. Meanwhile, a higher accumulation of H_2_O_2_ was detected with SA application under these stress conditions. H_2_O_2_, as a potent signaling molecule, has a dual role in plants in the regulation of plant growth, metabolism, and stress tolerance (Anjum et al., 2022). Also, it has been reported that abscisic acid-induced H_2_O_2_ accumulation enhances antioxidant capacity against Ca(NO_3_)_2_ stress (Shu et al., 2016). The increment in H_2_O_2_ content accompanied by unchanged EL under SA-pretreated Cd stress suggests that SA/H_2_O_2_ induces ROS-scavenging machinery and activates antioxidant defense in Caucasian wingnut.

As a result of drought stress and Cd toxicity, nutrient imbalance occurred, causing an increased concentration of P, K, Mg, and Ca in all the treatments (except P in Cd-stressed plants). Among micronutrients, Cu content was negatively affected by drought and Cd stress application. In contrast, Fe, Zn, and Mn tended to increase with stress conditions in Caucasian wingnut leaves. There are several studies in the literature that have similar or opposite findings to ours, for instance, studies on *Pfaffia glomerata* (Gomes et al., 2013), *Morus alba* (Guo et al., 2021), and *Brassica napus* (Dikšaitytė et al., 2023) under Cd stress and *Fagus sylvatica* (Peuke and Rennenberg, 2011) and *Ricinus communis* (Tadayyon et al., 2018) under drought stress. The results for mineral nutrient content in plants under stress conditions are contradictory due to the fact that the effects are highly dependent on plant species and experimental conditions (Mourato et al., 2019). In contrast, foliar application of SA showed a positive effect on the distribution of macro- and micronutrients in Caucasian wingnut, particularly at the Cd level under Cd stress alone and Cd+D treatments. Similar to our findings, SA pretreatment lowered Cd content in *Z. mays* (Gondor et al., 2016) and *Oryza sativa* (Wang et al., 2021).

Upregulation of the oxidative defense system is a key strategy in combating Cd or drought stress in plants (Hasanuzzaman et al., 2020). In this defense mechanism, APX, CAT, GR, POX, and SOD all fulfill specific roles, and the resulting reactions influence the homeostasis of ROS within the plant’s cells. This, in turn, affects the tolerance of plants to stressful conditions. SOD is known to catalyze the dismutation of toxic superoxide by converting it into H_2_O_2_ and oxygen. In addition, POX and CAT, located in the cytoplasm, and APX and GR, located in the chloroplasts, act to detoxify the H_2_O_2_ in plant cells (Liu et al., 2006). In this study, SOD activity increased in Caucasian wingnut in response to the combination of drought and Cd stresses. Similar conclusions have been reported in research on *Pisum sativum* under Cd stress (El-Amier et al., 2019), *Syzygium cumini* under water deficit (Zafar et al., 2021), *R. sativus* under cadmium and drought stress (Tuver et al., 2022), and *A. chilensis* under drought stress (González-Villagra et al., 2022). In the current study, we compared the effects of Cd and drought stress, both alone and in a combination of these two stress factors. In terms of antioxidant defense, Cd and Cd+D stress applications act similarly on the behavior of enzyme activities. POX, CAT, APX, and GR activities decreased with Cd treatments in Caucasian wingnut in this study. However, in spite of the decrease in those enzyme activities, leaf RWC, chlorophyll fluorescence content, and electrolyte leakage were maintained, and less reduction of osmotic potential and H_2_O_2_ was determined in this species under Cd toxicity compared to drought stress. To regulate this defense process, the activity of other enzymatic or non-enzymatic antioxidants may have been increased. Furthermore, variations in antioxidant enzyme activity may vary according to the extent of abiotic stress factors, the duration of exposure, and the developmental stage of the plants (Hasanuzzaman et al., 2020). In contrast, SA acted as a signaling molecule in this study, and foliar SA application improved the leaf antioxidant defense system and significantly increased GR activity compared to non-SA treatment in Caucasian wingnut subjected to cadmium stress, drought stress, and their combination. Similar to our findings, it was found that exogenous application of SA can help improve stress tolerance in *S. cumini* (Zafar et al., 2021) and *A. chilensis* (González-Villagra et al., 2022) under drought stress and *Cucumis melo* (Zhang et al., 2015) and *S. tuberosum* (Li et al., 2019) under Cd stress. These findings suggest that the foliar application of SA may help plants adapt to drought stress and Cd toxicity and may improve their ability to withstand the combined application of stress. Moreover, in previous studies, CAT activity was reported as inhibited with SA application (Klessig et al., 2000; Larkindale and Huang, 2004). These reports confirm the current results that the effect that SA application did not change CAT activity when Cd and drought stresses were applied alone, while it decreased CAT activity when Cd and drought stresses were applied in combination. From the findings of the study, we can conclude that drought stress alone negatively affected Caucasian wingnut when compared to drought stress applied in combination with Cd stress. Contrary to the results in most literature, cadmium stress application was not as detrimental as drought stress application. Additionally, here, we have presented a summary of the adaptive mechanisms demonstrated by the Caucasian wingnut in response to Cd toxicity, drought stress, and their combination with the exogenous application of SA. Regarding Caucasian wingnut, further studies are warranted in the future to better understand the effects of Cd toxicity on physiological and biochemical relations in the improvement of survival and phytoremediation potential of this woody species.

## 5 Conclusion

The research was conducted with the aim of evaluating the role of exogenous salicylic acid in increasing the tolerance of *P. fraxinifolia* L. toward cadmium toxicity, drought stress, and their combination through the observation of physiological traits and biochemical pathways, particularly the antioxidant defense system. Our study demonstrates that SA treatment can stimulate the antioxidant defense system; reduce lipid peroxidation, electrolyte leakage, and cadmium content; enhance leaf length, osmotic potential, relative water content, and hydrogen peroxide; and upregulate macro- and micronutrients in Caucasian wingnut plants grown under cadmium stress, drought stress, and the combination of these two stressors. The tolerance of Caucasian wingnut to cadmium seems to be high, suggesting that this plant may be a possible Cd accumulator. However, the amount of Cd concentration in other vegetative organs requires measurement, and further investigation is needed.

## Data availability statement

The raw data supporting the conclusions of this article will be made available by the authors, without undue reservation.

## Author contributions

HT: Conceptualization, Data curation, Investigation, Writing – original draft, Writing – review & editing, Formal analysis, Methodology. BC: Formal analysis, Methodology, Writing – review & editing. SS: Data curation, Methodology, Writing – review & editing. PP: Data curation, Methodology, Writing – review & editing.
