# Supplementary material for: Salicylic acid alleviates the effects of cadmium and drought stress by regulating water status, ions, and antioxidant defense in Pterocarya fraxinifolia
Source: Front Plant Sci. 2024 Jan 12;14:1339201. doi: 10.3389/fpls.2023.1339201 (PMC10811004; doi:10.3389/fpls.2023.1339201)
Supplement: Supplementary file 1 [file DataSheet_1.docx]

**Supplementary file**

**Table S1.** ANOVA results for leaf length, relative water content, water potential, quantum yield, electron leakage, thiobarbituric acid reactive substances and hydrogen peroxide with drought, Cd and salicylic acid as fixed factors.

| Factor | Df | Sumsq | MeanSQ | F | p | Factor | Df | Sumsq | MeanSQ | F | p |
| --- | --- | --- | --- | --- | --- | --- | --- | --- | --- | --- | --- |
| Leaf length | | | | | | EL | | | | | |
| Drought | 1 | 58.14 | 58.14 | 15.78 | **<0.001** | Drought | 1 | 0.04 | 0.04 | 0.018 | 0.9 |
| Cd | 1 | 1.38 | 1.38 | 0.375 | 0.54 | Cd | 1 | 10.4 | 10.4 | 4.428 | 0.05 |
| SA | 1 | 0.05 | 0.05 | 0.014 | 0.91 | SA | 1 | 64.03 | 64.03 | 27.26 | **<0.001** |
| Drought:Cd | 1 | 24.75 | 24.75 | 6.716 | **<0.05** | Drought:Cd | 1 | 1.31 | 1.31 | 0.556 | 0.47 |
| Drought:SA | 1 | 42.58 | 42.58 | 11.55 | **<0.01** | Drought:SA | 1 | 66 | 66 | 28.1 | **<0.001** |
| Cd:SA | 1 | 93.61 | 93.61 | 25.4 | **<0.001** | Cd:SA | 1 | 0.28 | 0.28 | 0.12 | 0.73 |
| Drought:Cd:SA | 1 | 29.43 | 29.43 | 7.986 | **<0.01** | Drought:Cd:SA | 1 | 1.13 | 1.13 | 0.48 | 0.5 |
| Residuals | 16 | 206.4 | 3.69 |  |  | Residuals | 16 | 37.59 | 2.35 |  |  |
| RWC | | | | | | TBARS | | | | | |
| Drought | 1 | 569.4 | 569.4 | 137.7 | **<0.001** | Drought | 1 | 237.5 | 237.5 | 119.7 | **<0.001** |
| Cd | 1 | 0 | 0 | 0.001 | 0.98 | Cd | 1 | 27.95 | 27.95 | 14.08 | **<0.01** |
| SA | 1 | 122 | 122 | 29.49 | **<0.001** | SA | 1 | 50.17 | 50.17 | 25.28 | **<0.001** |
| Drought:Cd | 1 | 0.2 | 0.2 | 0.044 | 0.84 | Drought:Cd | 1 | 28.38 | 28.38 | 14.3 | **<0.01** |
| Drought:SA | 1 | 0 | 0 | 0.005 | 0.94 | Drought:SA | 1 | 7.15 | 7.15 | 3.603 | 0.08 |
| Cd:SA | 1 | 32.9 | 32.9 | 7.957 | **<0.05** | Cd:SA | 1 | 0.02 | 0.02 | 0.01 | 0.92 |
| Drought:Cd:SA | 1 | 11.9 | 11.9 | 2.878 | 0.11 | Drought:Cd:SA | 1 | 13.65 | 13.65 | 6.878 | **<0.05** |
| Residuals | 16 | 66.2 | 4.1 |  |  | Residuals | 16 | 31.75 | 1.98 |  |  |
| WP | | | | | | H2O2 | | | | | |
| Drought | 1 | 1.175 | 1.175 | 28196 | **<0.001** | Drought | 1 | 8573 | 8573 | 5487 | **<0.001** |
| Cd | 1 | 0.014 | 0.014 | 324.9 | **<0.001** | Cd | 1 | 151 | 151 | 96.64 | **<0.001** |
| SA | 1 | 0.051 | 0.051 | 1232 | **<0.001** | SA | 1 | 2332 | 2332 | 1493 | **<0.001** |
| Drought:Cd | 1 | 0.048 | 0.048 | 1145 | **<0.001** | Drought:Cd | 1 | 651 | 651 | 416.7 | **<0.001** |
| Drought:SA | 1 | 0.008 | 0.008 | 202.5 | **<0.001** | Drought:SA | 1 | 7 | 7 | 4.234 | 0.06 |
| Cd:SA | 1 | 0.048 | 0.048 | 1145 | **<0.001** | Cd:SA | 1 | 657 | 657 | 420.7 | **<0.001** |
| Drought:Cd:SA | 1 | 3E-04 | 3E-04 | 8.1 | 0.01 | Drought:Cd:SA | 1 | 874 | 874 | 559.1 | **<0.001** |
| Residuals | 16 | 7E-04 | 0 |  |  | Residuals | 16 | 25 | 2 |  |  |
| Fv/Fm | | | | | |  |  |  |  |  |  |
| Drought | 1 | 0.011 | 0.011 | 51.77 | **<0.001** |  |  |  |  |  |  |
| Cd | 1 | 0.004 | 0.004 | 17.5 | **<0.001** |  |  |  |  |  |  |
| SA | 1 | 4E-04 | 4E-04 | 1.978 | 0.17 |  |  |  |  |  |  |
| Drought:Cd | 1 | 0.013 | 0.013 | 58.5 | **<0.001** |  |  |  |  |  |  |
| Drought:SA | 1 | 0.002 | 0.002 | 7.068 | **<0.05** |  |  |  |  |  |  |
| Cd:SA | 1 | 6E-04 | 6E-04 | 2.818 | 0.1 |  |  |  |  |  |  |
| Drought:Cd:SA | 1 | 0.002 | 0.002 | 7.164 | **<0.05** |  |  |  |  |  |  |
| Residuals | 16 | 0.01 | 2E-04 |  |  |  |  |  |  |  |  |

**Table S2.** ANOVA results for leaf nutrients P, Mg, Zn, K, Fe, Mn, Ca, Cu and Cd with drought, cadmium and salicylic acid as fixed factors.

| Factor | Df | Sumsq | MeanSQ | F | p | Factor | Df | Sumsq | MeanSQ | F | p | Factor | Df | Sumsq | MeanSQ | F | p |
| --- | --- | --- | --- | --- | --- | --- | --- | --- | --- | --- | --- | --- | --- | --- | --- | --- | --- |
| P | | | | | | Mg | | | | | | Zn | | | | | |
| Drought | 1 | 738034 | 738034 | 221187 | **<0.001** | Drought | 1 | 6761 | 6761 | 21084 | **<0.001** | Drought | 1 | 23.622 | 23.622 | 404940 | **<0.001** |
| Cd | 1 | 292379 | 292379 | 87625 | **<0.001** | Cd | 1 | 398376 | 398376 | 1E+06 | **<0.001** | Cd | 1 | 3.832 | 3.832 | 65692 | **<0.001** |
| SA | 1 | 1.8E+07 | 1.8E+07 | 5E+06 | **<0.001** | SA | 1 | 125121 | 125121 | 390170 | **<0.001** | SA | 1 | 2.933 | 2.933 | 50280 | **<0.001** |
| Drought:Cd | 1 | 1272371 | 1272371 | 381326 | **<0.001** | Drought:Cd | 1 | 101030 | 101030 | 315045 | **<0.001** | Drought:Cd | 1 | 1.211 | 1.211 | 20752 | **<0.001** |
| Drought:SA | 1 | 5159256 | 5159256 | 2E+06 | **<0.001** | Drought:SA | 1 | 326077 | 326077 | 1E+06 | **<0.001** | Drought:SA | 1 | 1.321 | 1.321 | 22641 | **<0.001** |
| Cd:SA | 1 | 515475 | 515475 | 154486 | **<0.001** | Cd:SA | 1 | 108220 | 108220 | 337468 | **<0.001** | Cd:SA | 1 | 2.202 | 2.202 | 37752 | **<0.001** |
| Drought:Cd:SA | 1 | 4579219 | 4579219 | 1E+06 | **<0.001** | Drought:Cd:SA | 1 | 2E+06 | 1759491 | 5E+06 | **<0.001** | Drought:Cd:SA | 1 | 2.555 | 2.555 | 43792 | **<0.001** |
| Residuals | 16 | 53 | 3 |  |  | Residuals | 16 | 5 | 0 |  |  | Residuals | 16 | 0.001 | 0 |  |  |
| K | | | | | | Fe | | | | | | Mn | | | | | |
| Drought | 1 | 5.9E+07 | 5.9E+07 | 39.314 | **<0.001** | Drought | 1 | 28.908 | 28.908 | 115633 | **<0.001** | Drought | 1 | 1.4162 | 1.4162 | 17888.9 | **<0.001** |
| Cd | 1 | 249943 | 249943 | 0.167 | 0.6885 | Cd | 1 | 2.627 | 2.627 | 10507 | **<0.001** | Cd | 1 | 0.1751 | 0.1751 | 2211.84 | **<0.001** |
| SA | 1 | 5.2E+07 | 5.2E+07 | 34.9 | **<0.001** | SA | 1 | 1.804 | 1.804 | 7216.1 | **<0.001** | SA | 1 | 0.0002 | 0.0002 | 2.579 | 0.128 |
| Drought:Cd | 1 | 5.1E+07 | 5.1E+07 | 33.891 | **<0.001** | Drought:Cd | 1 | 6.784 | 6.784 | 27136 | **<0.001** | Drought:Cd | 1 | 2.2387 | 2.2387 | 28278.4 | **<0.001** |
| Drought:SA | 1 | 4665630 | 4665630 | 3.112 | 0.0968 | Drought:SA | 1 | 1.363 | 1.363 | 5453.1 | **<0.001** | Drought:SA | 1 | 0.306 | 0.306 | 3865.32 | **<0.001** |
| Cd:SA | 1 | 1.9E+07 | 1.9E+07 | 12.924 | **<0.01** | Cd:SA | 1 | 2.018 | 2.018 | 8073.6 | **<0.001** | Cd:SA | 1 | 0.1335 | 0.1335 | 1686.37 | **<0.001** |
| Drought:Cd:SA | 1 | 7093904 | 7093904 | 4.731 | **<0.05** | Drought:Cd:SA | 1 | 0.115 | 0.115 | 459.3 | **<0.001** | Drought:Cd:SA | 1 | 0.0057 | 0.0057 | 72.053 | **<0.001** |
| Residuals | 16 | 2.4E+07 | 1499359 |  |  | Residuals | 16 | 0.004 | 0 |  |  | Residuals | 16 | 0.0013 | 0.0001 |  |  |
| Ca | | | | | | Cu | | | | | | Cd | | | | | |
| Drought | 1 | 1.5E+07 | 1.5E+07 | 291671 | **<0.001** | Drought | 1 | 0.0003 | 0.00027 | 16 | **<0.01** | Drought | 1 | 0.0015 | 0.0015 | 193.2 | **<0.001** |
| Cd | 1 | 187924 | 187924 | 3675 | **<0.001** | Cd | 1 | 0.0014 | 0.00135 | 81 | **<0.001** | Cd | 1 | 1.8676 | 1.8676 | 247641 | **<0.001** |
| SA | 1 | 2.9E+07 | 2.9E+07 | 560956 | **<0.001** | SA | 1 | 0.0038 | 0.00375 | 225 | **<0.001** | SA | 1 | 0.0133 | 0.0133 | 1763.7 | **<0.001** |
| Drought:Cd | 1 | 211365 | 211365 | 4133 | **<0.001** | Drought:Cd | 1 | 0.0017 | 0.00167 | 100 | **<0.001** | Drought:Cd | 1 | 0.0015 | 0.0015 | 193.2 | **<0.001** |
| Drought:SA | 1 | 1606424 | 1606424 | 31413 | **<0.001** | Drought:SA | 1 | 0.0006 | 0.0006 | 36 | **<0.001** | Drought:SA | 1 | 0.0072 | 0.0072 | 951.5 | **<0.001** |
| Cd:SA | 1 | 5297258 | 5297258 | 103585 | **<0.001** | Cd:SA | 1 | 0.0004 | 0.00042 | 25 | **<0.001** | Cd:SA | 1 | 0.0133 | 0.0133 | 1763.7 | **<0.001** |
| Drought:Cd:SA | 1 | 2.6E+07 | 2.6E+07 | 501769 | **<0.001** | Drought:Cd:SA | 1 | 7E-05 | 6.7E-05 | 4 | 0.0628 | Drought:Cd:SA | 1 | 0.0072 | 0.0072 | 951.5 | **<0.001** |
| Residuals | 16 | 818 | 51 |  |  | Residuals | 16 | 0.0003 | 1.7E-05 |  |  | Residuals | 16 | 0.0001 | 0 |  |  |

**Table S3.** ANOVA results for catalase, ascorbate peroxidase, glutathione reductase and superoxide dismutase, with drought, Cd and salicylic acid as fixed factors.

| Factor | Df | Sumsq | MeanSQ | F | p | Factor | Df | Sumsq | MeanSQ | F | p |
| --- | --- | --- | --- | --- | --- | --- | --- | --- | --- | --- | --- |
| CAT | | | | | | GR | | | | | |
| Drought | 1 | 0.0034 | 0.0034 | 16.584 | **<0.001** | Drought | 1 | 0.019 | 0.019 | 3.506 | 0.0734 |
| Cd | 1 | 0.0005 | 0.0005 | 2.574 | 0.1217 | Cd | 1 | 0.0924 | 0.0924 | 17.048 | **<0.001** |
| SA | 1 | 0.0069 | 0.0069 | 33.64 | **<0.001** | SA | 1 | 0.495 | 0.495 | 91.282 | **<0.001** |
| Drought:Cd | 1 | 0.0026 | 0.0026 | 12.807 | **<0.01** | Drought:Cd | 1 | 0.1568 | 0.1568 | 28.914 | **<0.001** |
| Drought:SA | 1 | 0.0004 | 0.0004 | 1.843 | 0.1873 | Drought:SA | 1 | 0.1128 | 0.1128 | 20.803 | **<0.001** |
| Cd:SA | 1 | 0.0002 | 0.0002 | 0.746 | 0.3962 | Cd:SA | 1 | 0.1058 | 0.1058 | 19.51 | **<0.001** |
| Drought:Cd:SA | 1 | 0.0023 | 0.0023 | 11.102 | **<0.01** | Drought:Cd:SA | 1 | 0.0841 | 0.0841 | 15.499 | **<0.001** |
| Residuals | 16 | 0.0049 | 0.0002 |  |  | Residuals | 16 | 0.1302 | 0.0054 |  |  |
| APX | | | | | | SOD | | | | | |
| Drought | 1 | 324.5 | 324.5 | 110.77 | **<0.001** | Drought | 1 | 9.67 | 9.67 | 0.909 | 0.3498 |
| Cd | 1 | 565.2 | 565.2 | 192.93 | **<0.001** | Cd | 1 | 2.43 | 2.43 | 0.228 | 0.6373 |
| SA | 1 | 1168.1 | 1168.1 | 398.77 | **<0.001** | SA | 1 | 160.7 | 160.7 | 15.11 | **<0.001** |
| Drought:Cd | 1 | 14.3 | 14.3 | 4.867 | **<0.05** | Drought:Cd | 1 | 107.64 | 107.64 | 10.121 | **<0.001** |
| Drought:SA | 1 | 389.6 | 389.6 | 133.01 | **<0.001** | Drought:SA | 1 | 26.23 | 26.23 | 2.466 | 0.1294 |
| Cd:SA | 1 | 6.7 | 6.7 | 2.286 | 0.1436 | Cd:SA | 1 | 6.56 | 6.56 | 0.617 | 0.4399 |
| Drought:Cd:SA | 1 | 7.3 | 7.3 | 2.504 | 0.1267 | Drought:Cd:SA | 1 | 58.29 | 58.29 | 5.481 | **<0.05** |
| Residuals | 16 | 70.3 | 2.9 |  |  | Residuals | 16 | 255.24 | 10.63 |  |  |
